# The effect of hyperbaric oxygen therapy on the clinical outcomes of necrotizing soft tissue infections: a systematic review and meta-analysis

**DOI:** 10.1186/s13017-023-00490-y

**Published:** 2023-03-25

**Authors:** Chengzi Huang, Yilian Zhong, Chaochi Yue, Bin He, Yaling Li, Jun Li

**Affiliations:** 1 Department of Anorectum, The Affiliated Hospital of Southwest Medical University, Luzhou, China; 2 Department of Pharmacy, The Affiliated Hospital of Southwest Medical University, Luzhou, China

**Keywords:** Necrotizing fasciitis, Necrotizing soft tissue infection, Fournier gangrene, Mortality, Complication, Hyperbaric oxygen therapy

## Abstract

**Background:**

To determine the efficacy of hyperbaric oxygen therapy (HBO) in the treatment of necrotizing soft tissue infections (NSTI), we conducted a meta-analysis of the available evidence.

**Methods:**

Data sources were PubMed, Embase, Web of Science, Cochrane Library, and reference lists. The study included observational trials that compared HBO with non-HBO, or standard care. The primary outcome was the mortality rate. Secondary outcomes were the number of debridement, amputation rate and complication rate. Relative risks or standardized mean differences with 95% confidence intervals were calculated for dichotomous and continuous outcomes, respectively.

**Results:**

A total of retrospective cohort and case-control studies were included, including 49,152 patients, 1448 who received HBO and 47,704 in control. The mortality rate in the HBO group was significantly lower than that in the non-HBO group [RR = 0.522, 95% CI (0.403, 0.677), *p* < 0.05]. However, the number of debridements performed in the HBO group was higher than in the non-HBO group [SMD = 0.611, 95% CI (0.012, 1.211), *p* < 0.05]. There was no significant difference in amputation rates between the two groups [RR = 0.836, 95% CI (0.619, 1.129),* p* > 0.05]. In terms of complications, the incidence of MODS was lower in the HBO group than in the non-HBO group [RR = 0.205, 95% CI (0.164, 0.256),* p* < 0.05]. There was no significant difference in the incidence of other complications, such as sepsis, shock, myocardial infarction, pulmonary embolism, and pneumonia, between the two groups (*p* > 0.05).

**Conclusion:**

The current evidence suggests that the use of HBO in the treatment of NSTI can significantly reduce the mortality rates and the incidence rates of complications. However, due to the retrospective nature of the studies, the evidence is weak, and further research is needed to establish its efficacy. It is also important to note that HBO is not available in all hospitals, and its use should be carefully considered based on the patient's individual circumstances. Additionally, it is still worthwhile to stress the significance of promptly evaluating surgical risks to prevent missing the optimal treatment time.

**Supplementary Information:**

The online version contains supplementary material available at 10.1186/s13017-023-00490-y.

## Introduction

Necrotizing soft tissue infections (NSTI), also known as necrotizing fasciitis (NF), are a rare but serious type of infection that can rapidly progress and lead to life-threatening consequences if not promptly and aggressively treated [1–4]. NSTI can be secondary to any skin injury or blood-borne transmission, such as postoperative skin biopsy, lacerated wounds, insect bites, pricking wounds, burns, surgical wounds, skin abscesses, herpes zoster, and venous ulceration [5, 6]. Due to the inconsistency between the early local symptoms and the systemic symptoms and the lack of specificity in the clinical presentation, NSTI is easily misdiagnosed in clinical practice. The early stages of NSTI may not be evident, but the condition can deteriorate rapidly within hours. The major systemic symptoms can include sustained fever, tachycardia, insufficient circulatory volume, hypoproteinemia, electrolyte disturbances, hyperglycemia, etc. If treatment is not timely, it can lead to septicemia, infectious shock, multiple organ dysfunction syndrome (MODS) or even death [2, 3]. Regardless of the underlying cause, NSTI demands prompt and comprehensive surgical removal of damaged tissue, antibiotics that are effective against a wide range of bacteria, and intense supportive care [7]. NSTI differs from other soft tissue infections in that it can spread quickly through the subcutaneous tissue and fascia and has a high mortality rate, which has been estimated to be between 20 and 30%, or even higher [8–10]. Given the high mortality rate of NSTI, the use of effective adjuvant therapies to improve treatment outcomes is warranted. Hyperbaric oxygen therapy (HBO) is one of these modalities [11].

HBO has been used to treat various conditions for over 50 years, starting with Brummelkamp's finding that hyperbaric oxygen conditions can suppress anaerobic infections [12]. HBO has a bacteria-killing effect on anaerobic infections and has been demonstrated to improve tissue perfusion, promote angiogenesis, increase the oxygen level in tissues, and inhibit toxin production [13, 14]. It has also been used to treat mixed infections, including NSTI. The high-oxygen environment created by HBO can act as a barrier to prevent the spread of infection in NSTI [7, 15]. An expert consensus from China recommends HBO as an adjunctive therapy due to its ability to improve oxygen delivery to local tissues and increase survival rates, and provide favorable conditions for wound healing [16]. However, some societies such as the Infectious Disease Society of America recommend against its use [10]. An international multi-society document of skin and soft-tissue infections (SSTIs) in 2022 points that the role of HBO as an adjunctive treatment has been debated. There is currently no valid research evidence or published prospective randomized clinical trials (RCTs) that examine the impact of HBO on wound healing [11]. Therefore, research progress on NSTI has become extremely significant, and close attention should be paid.

Given the rarity and seriousness of NSTI, and the absence of evidence-based guidance on using HBO in its treatment, we carried out a systematic review and meta-analysis to assess the impact of HBO on the clinical outcomes of NSTI and provide evidence-based guidance for its use in this condition.

## Methods

This systematic review and meta-analysis followed the Preferred Reporting Items for Systematic reviews and Meta-Analyses (PRISMA) guidelines [17] (The PRISMA 2020 Checklist and  PRISMA 2020 for Abstracts Checklist were showed in Additional file 1, 2). The review protocol was registered in INPLASY register (INPLASY202320119).

### Search strategy

A literature search was conducted using PubMed, Embase, Web of Science, and the Cochrane Database of Systematic Reviews from their inception to November 28, 2022 to identify relevant studies on the use of HBO in the treatment of NSTI, including NF and Fournier gangrene (FG). The search terms used were "necrotizing soft tissue infection," "necrotizing fasciitis," "Fournier gangrene," and "hyperbaric oxygen therapy." The language of the studies included in the review were restricted to English. The literature search strategy and full search string can be found in Additional file 3: Appendix A.

### Selection criteria

The following criteria were used to determine eligibility for inclusion in this study: (1) Clinical trials and observational studies published before November 28, 2022; (2) Participants diagnosed with NSTI (or NF or FG); (3) Studies that compared the use of HBO with no use of HBO; (4) Studies that reported at least one outcome of interest. The following types of studies were excluded: (1) Conference abstracts, reviews, animal studies, case reports, editorials, letters, etc.; (2) Duplicate studies; (2) Full text unavailable; (3) Studies from which data could not be extracted; (4) Studies with inappropriate outcomes; (5) Studies with low sample sizes (total n < 10). Two reviewers (CH and YZ) independently reviewed candidate studies by screening title and abstract, and identified the studies which met the inclusion criteria. In the event of uncertainty, the eligibility of a study was discussed between the two reviewers (CH and YZ), and any disagreements were resolved depending on the third independent reviewer (BH).

### Data extraction

The following data were extracted from included studies, if available: first author, year of publication, study design, country or region of the study, sample size, mean or median age, sex, body regions affected, confounders and the outcome of interest.

### Statistical analysis

#### Qualitative synthesis

Two reviewers independently evaluated the characteristics and quality of the included studies using the Newcastle Ottawa Scale [18]. Any discrepancies were resolved through discussion and further review.

#### Quantitative synthesis

Relative risks (RRs) or standardized mean differences (SMDs) with 95% confidence intervals (CIs) were calculated for dichotomous and continuous outcomes, respectively. As clinical heterogeneity and methodological heterogeneity are inevitable at any time, we performed a meta-analysis using a random effect model.

#### Sources of bias

Publication bias was evaluated by visual inspection of funnel plots.

#### Subgroup analyses

Subgroup analyses were conducted based on pathological entity.

#### Statistical software

All statistical analyses were carried out using R software (version 4.0.2). A *p* value ≤ 0.05 was considered statistically significant.

#### Evidence certainty

The Grading of Recommendations Assessment, Development and Evaluation (GRADE) system was used to access the overall certainty of evidence. By GRADE system, the certainty of evidence derived from cohort studies receive an initial grade of low quality. The quality of evidence from cohort studies can be improved at larger effect sizes (RR ≥ 2 or ≤ 0. 5), dose–response gradients, or attenuation by plausible confounding after excluding various factors that could lead to downgrading. Finally, the evidence of outcomes can be graded as being of high, moderate, low, or very low.

## Results

### Data extraction and quality assessment

#### Systematic review process

**Fig. 1:**
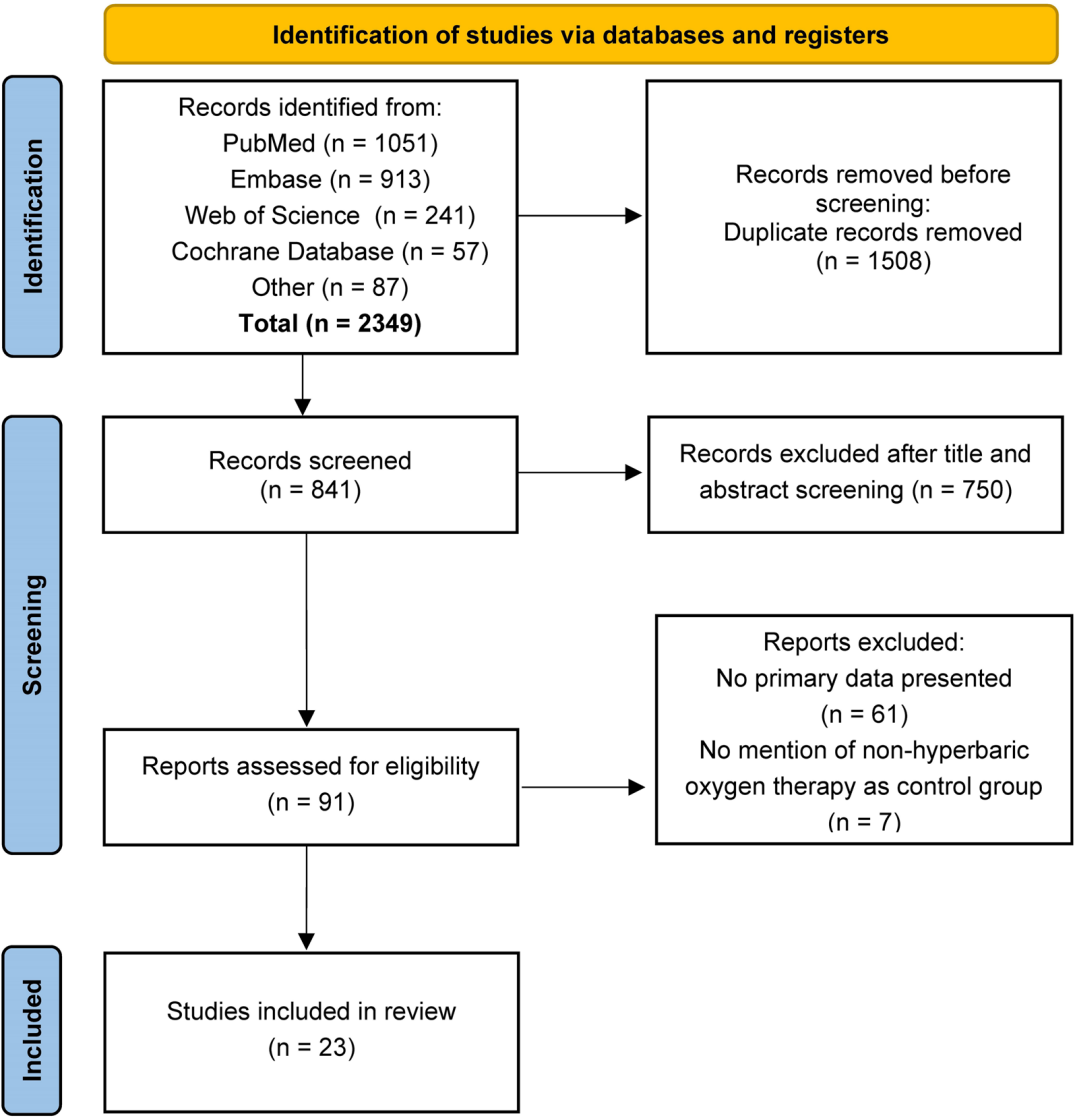
Flow chart of the selection of publications included in the meta-analysis

A literature search identified a total of 2349 studies, of which 1508 were removed due to duplication or overlap. An additional 750 studies were excluded after screening titles and abstracts, leaving 91 full-text studies. Of these, 68 studies that did not meet the inclusion criteria were excluded, leaving 23 studies that were eligible for inclusion in the review. Figure 1 shows a flow chart illustrating the process of selecting publications for inclusion.

#### Quality assessment

The Newcastle–Ottawa quality assessment scale was used to evaluate the quality of the evidence. According to this scale, all of the selected studies received at least 5 stars, indicating a low to moderate risk of bias (Table 1).

**Table 1:** Newcastle-Ottawa Scale for risk of bias assessment of studies included in the meta-analysis

Study	Selection				Comparability	Outcome			Overall
	Representativeness of exposed cohort	Selection of nonexposed	Ascertainment of exposure	Outcome not present at start		Assessment of outcome	Adequate follow-up length	Adequacy of follow-up	
Mladenov (3)	★	★	★	★	★	★	★	★	8
Tutino (19)	★	★	★	★	★	★	☆	★	7
Omar (20)	★	★	★	★	★	★	★	☆	7
Creta (21)	★	★	★	★	★	★	☆	★	7
Anheuser (7)	★	★	★	★	☆	☆	★	☆	5
Thrane (22)	★	★	★	★	★	☆	★	☆	6
Devaney (23)	★	★	★	☆	☆	★	☆	★	5
Hung (24)	★	★	★	★	☆	★	★	★	7
Li (25)	★	★	★	☆	☆	★	★	☆	5
Shaw (26)	★	★	★	★	☆	★	★	☆	6
Chai (27)	★	★	★	★	★★	★	★	★	9
Massey (28)	★	★	☆	★	☆	★	☆	★	5
Hassan (29)	★	★	★	★	★	★	☆	☆	6
Mehl (30)	★	★	☆	★	☆	★	★	☆	5
George (31)	★	★	★	★	☆	★	★	☆	6
Krenk (32)	★	★	☆	★	☆	☆	★	★	5
Steven (33)	★	★	★	★	★	★	★	★	8
Wilkinson (34)	★	☆	★	★	☆	★	★	☆	5
Dahm (35)	★	★	★	☆	★	☆	★	★	6
Hollabaugh (36)	★	★	★	★	★	★	★	★	8
Shupak (37)	★	★	★	★	☆	★	★	★	7
Brown (38)	★	★	★	★	☆	★	★	☆	6
Riseman (39)	★	★	★	★	★	★	☆	★	7

☆, zero score; ★, one score; ★★, two scores

#### Characteristics of eligible studies

All 23 studies [3, 7, 19–39] included were retrospective studies with a total of 49,152 patients, and 1448 patients were treated HBO, versus 47,704 patients treated without HBO. Ten studies came from North America (8 from USA, 2 from Canada). Six studies came from Europe (2 from Germany, 2 from Italy, 2 from Denmark). Four studies came from Asia (1 from China, 1 from Taiwan, 1 from Singapore, and 1 from Israel). Two studies came from South America (Brazil), and only 1 study came from Oceania (Australia). 65.2% of the patients were males. The body regions infected varied among the studies, and the major body regions affected were head and neck, truncal, perianal, anorectal, perineal and genital areas. The primary outcome included the mortality rate. The secondary outcomes included the amputation rate, the number of debridement and complications. The complications in this meta-analysis were sepsis, shock, myocardial infarction, pulmonary embolism, pneumonia and MODS. The characteristics of the included studies are summarized in Table 2.

**Table 2:** Characteristics of the studies and patients included in the meta-analysis

Author	Year	Country/Region	Study type	Sample size			Sex, male, n (%)		Age(year)		Body regions affected	Confounders adjusted	Outcomes
				Total	HBO	Non-HBO	HBO	Non-HBO	HBO	Non-HBO			
Mladenov (3)	2022	Germany	Retrospective study	181	83	98	48 (57.8)	69 (70.4)	58.8^a^	64.3^a^	Lower extremity/perianal/genital/gluteal	Female, age, problem localization, comorbidities, LRINEC	[1] [2] [3] [4a, 4c]
Tutino (19)	2022	Italy	Retrospective study	23	13	10	16(69.6)		62.7^a^		Gluteal/inguinal/perineal/scrotal	Age, sex, BMI, comorbidities, ASA score, delay from symptoms to admission	[1]
Omar (20)	2021	Brazil	Retrospective comparative study	197	79	118	53 (67.0)	103 (87.2)	48.2^a^	46.6^a^	Perineal/scrotal/penile/perianal/gluteal/inguinal/abdominal	Age, sex, comorbidities	[1]
Creta (21)	2020	Italy	Retrospective observational case–control study	161	72	89	65 (90.3)	87 (97.8)	66.5 ± 15.2^a^		Genital	Age, gender, FGSI Score	[1]
Anheuser (7)	2018	Germany	Retrospective observational study	62	17	45	17 (100)	45 (100)	58^a^	60^a^	Scrotal/perineal/penile/rectal/inguinal/urethral/renal	Sex, age, comorbidities, clinical symptoms, laboratory and microbiological data	[1] [2] [4a, 4b]
Thrane (22)	2017	Denmark	Retrospective cohort study	43	30	13	15(50.0)	10(76.9)	55^b^	52^b^	Head and neck	Age, primary infectious focus, gender, presence of comorbidity	[1] [4b]
Devaney (23)	2015	US	Retrospective case-controlled study	341	275	66	158 (57.5)	50(75.7)	52.2^a^	55.7^a^	Head and neck/perineal/abdominal/truncal	Age, obesity, smoking, diabetes, comorbidities, Illness severity (LRINEC, APACHE III), organism	[1] [2] [3]
Hung (24)	2015	Taiwan	Retrospective study	60	12	48	50(83.3)		59.6 ± 14.5^a^		Dermatological/anorectal/urogenital	Age, severity, location	[1]
Li (25)	2015	China	Retrospective study	28	16	12	28(100)		46.13 ± 13.11^a^	48.42 ± 15.31^a^	Scrotal/perineal/perianal	Age, FGSI score, predisposing factors	[1] [2]
Shaw (26)	2014	USA	Retrospective cohort study	1583	117	1466	83(70.9)	1433(97.7)	56^b^	54^b^	△	Severity of illness, age, sex, race, comorbidities	[1]
Chai (27)	2012	Singapore	Retrospective study	45,913	405	45,508	243 (60.0)	29,612 (65.1)	54.6^a^	53.7^a^	Truncal/lower extremity	Age, gender, the patient’s county of residence, hospital characteristics (bed size, location and teaching status), the Deyo clinical co-morbidity index	[1] [3] [4a, 4b, 4c, 4d, 4e, 4f]
Massey (28)	2012	USA	Retrospective cohort study	80	32	48	19 (59.4)	24 (50.0)	55^a^	54^a^	△	Age, race, sex, affected site, BMI, comorbidity	[1] [3]
Hassan (29)	2010	USA	Retrospective chart review	67	29	38	18 (62.1)	20 (52.6)	49.6 ± 15.6^a^	50.7 ± 13.2^a^	△	Sex, race, etiology, smoker, alcohol user, complicated admission, comorbidity, diabetes mellitus	[1] [2] [3]
Mehl (30)	2010	Brazil	Retrospective study	40	26	14	31(77.5)		47.2^a^		Perineal/scrotal/thigh/inguinal/perianal/lumbosacral/penile/buttock/abdominal/lower extremity	Gender, age, location, predisposing factors, etiology, lesion location, laboratory tests and imaging, surgical procedures, antibiotics	[1]
George (31)	2009	Canada	Retrospective medical record review	78	48	30	49(62.8)		49.5 ± 19.6^a^		△	Age, immunosuppression, hypotension, truncal involvement, clostridium infection	[1] [2]
Krenk (32)	2007	Denmark	Retrospective study	19	11	8	3(27.2)	2(25.0)	59.5^a^	54.4^a^	Head and neck	Age, sex, etiological focus, bacteriology, comorbidity	[1]
Steven (33)	2005	USA	Retrospectively reviewed	42	26	16	24(92.3)	14(87.5)	57 ± 14^a^	57 ± 15^a^	Genital/scrotal	Age, race, comorbidity, BMI	[1] [4a, 4c, 4d, 4e, 4f]
Wilkinson (34)	2004	Australia	Retrospective cohort study	44	33	11	△		△		△	Age, race, comorbidity, site, etiology, symptom to hospital admission,	[1] [3]
Dahm (35)	2000	USA	Retrospective study	44	38	6	44(100)		56.3^a^		Anorectal/genital	Age, history of diabetes or chronic alcoholism, white blood cell count on admission, results of blood cultures, source of infection, extent and depth of the infection	[1]
Hollabaugh (36)	1998	USA	Retrospective study	26	14	12	26(100)		57^b^		Penile/perineal/periurethral	No report	[1]
Shupak (37)	1995	Israel	Retrospective study	37	25	12	14(56.0)	9(75.0)	52.9 ± 15^a^	57.4 ± 16^a^	△	Age, sex	[1] [2]
Brown (38)	1994	Canada	Retrospective study	54	30	24	22(73.3)	13(54.2)	51.3 ± 17.1^a^	61.6 ± 12.6^a^	Truncal	Age, sex, APACHE II score	[1] [2]
Riseman (39)	1990	USA	Retrospective study	29	17	12	11(64.7)	7(58.3)	68.5^a^	59.7^a^	Perineal/truncal	Age, sex, race, wound bacteriology, presence or absence of diabetes mellitus, peripheral vascular disease, obesity, antecedent trauma	[1]

a, Mean age, years; b, Median age, years(SD); △, not available; NF, necrotizing fasciitis; FG, Fournier’ s gangrene; NSTI, necrotizing soft tissue infection; HBO, hyperbaric oxygen therapy;[1], mortality rate; [2], the number of debridement; [3], amputation rate; [4], complications (4a, sepsis; 4b, shock; 4c, myocardial infarction; 4d, pulmonary embolism; 4e, MODS; 4f, pneumonia). LRINEC, Laboratory Risk Indicator for NEC rotating fasciitis; BMI, body mass index; ASA, American Society of Anesthesiologists; FGSI, Fournier gangrene severity index; APACHE, Acute Physiology and Chronic Health Evaluation

### Evidence synthesis

#### Primary outcomes

**Fig. 2:**
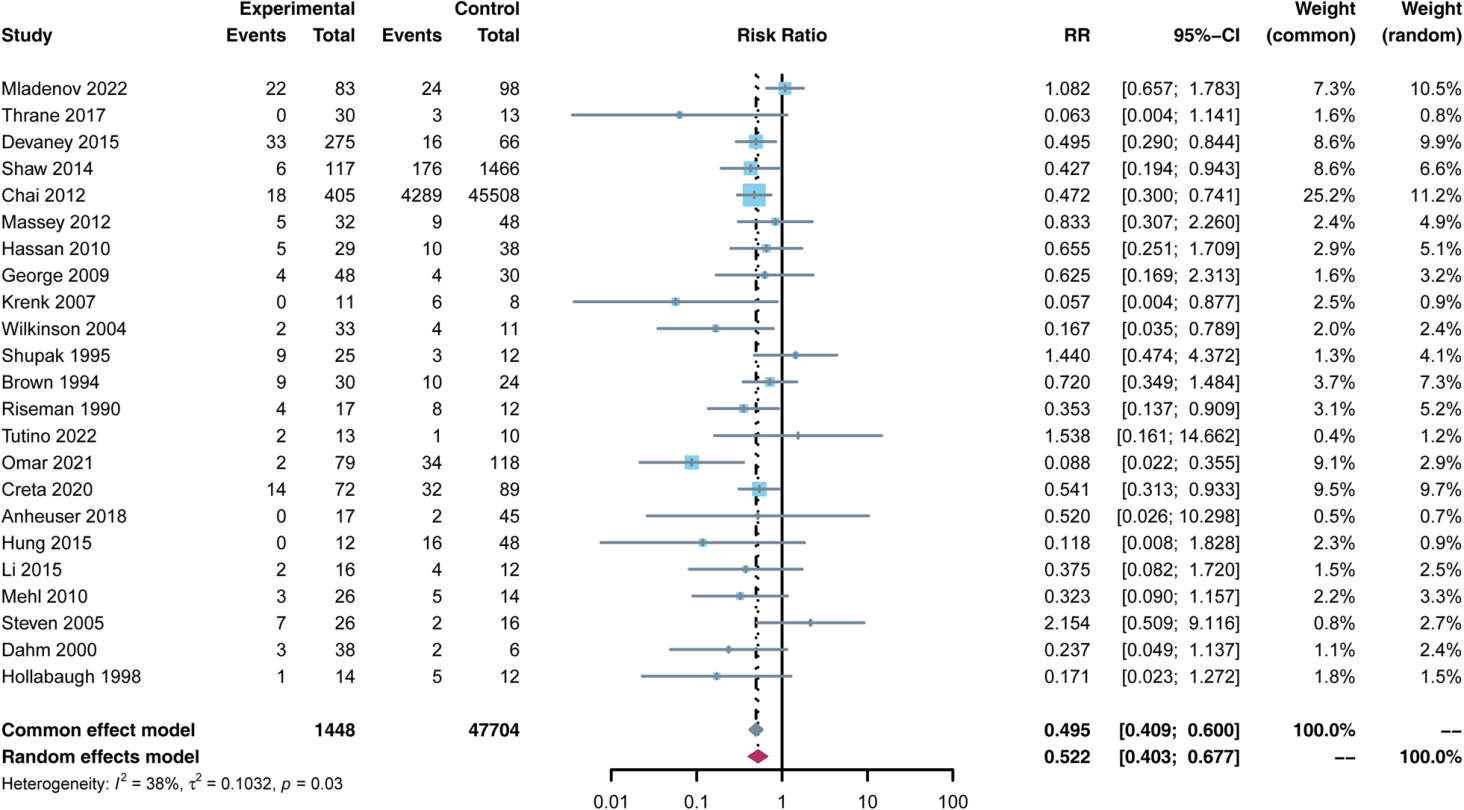
Forest plot of the mortality rate

As the primary outcome, the mortality rate was reported in all included studies. The mean mortality rate in the HBO group was 10.6% [95% CI (6.7, 14.5)] and the mean mortality rate in the non-HBO group was 25.6% [95% CI (19.5, 31.7)]. The study found that the mortality rate in the HBO group was significantly lower than that in the non-HBO group [RR = 0.522, 95% CI (0.403, 0.677), *p* < 0.05] (Fig. 2).

#### Secondary outcomes

**Fig. 3:**
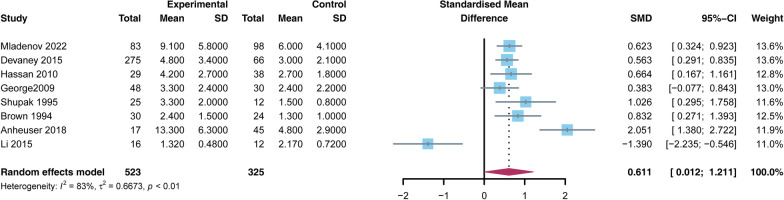
Forest plot of the number of debridement

The number of debridements was reported in 8 studies [3, 7, 23, 25, 29, 31, 37, 38]. The study found that the number of debridements in the HBO group was higher than in the non-HBO group [SMD = 0.611, 95% CI (0.012, 1.211), *p* < 0.05] (Fig. 3).

**Fig. 4:**
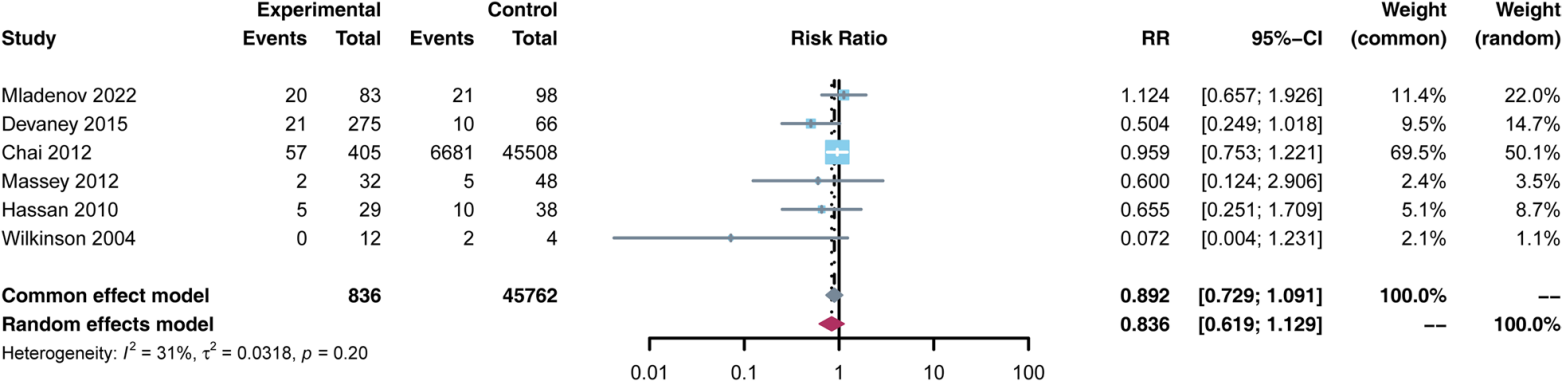
Forest plot of the amputation rate

The amputation rate was reported in 6 studies [3, 23, 27–29, 34]. The study found no statistical significance in the amputation rate between the HBO group and non-HBO group [RR = 0.836, 95% CI (0.619, 1.129), *p* > 0.05] (Fig. 4).

**Fig. 5:**
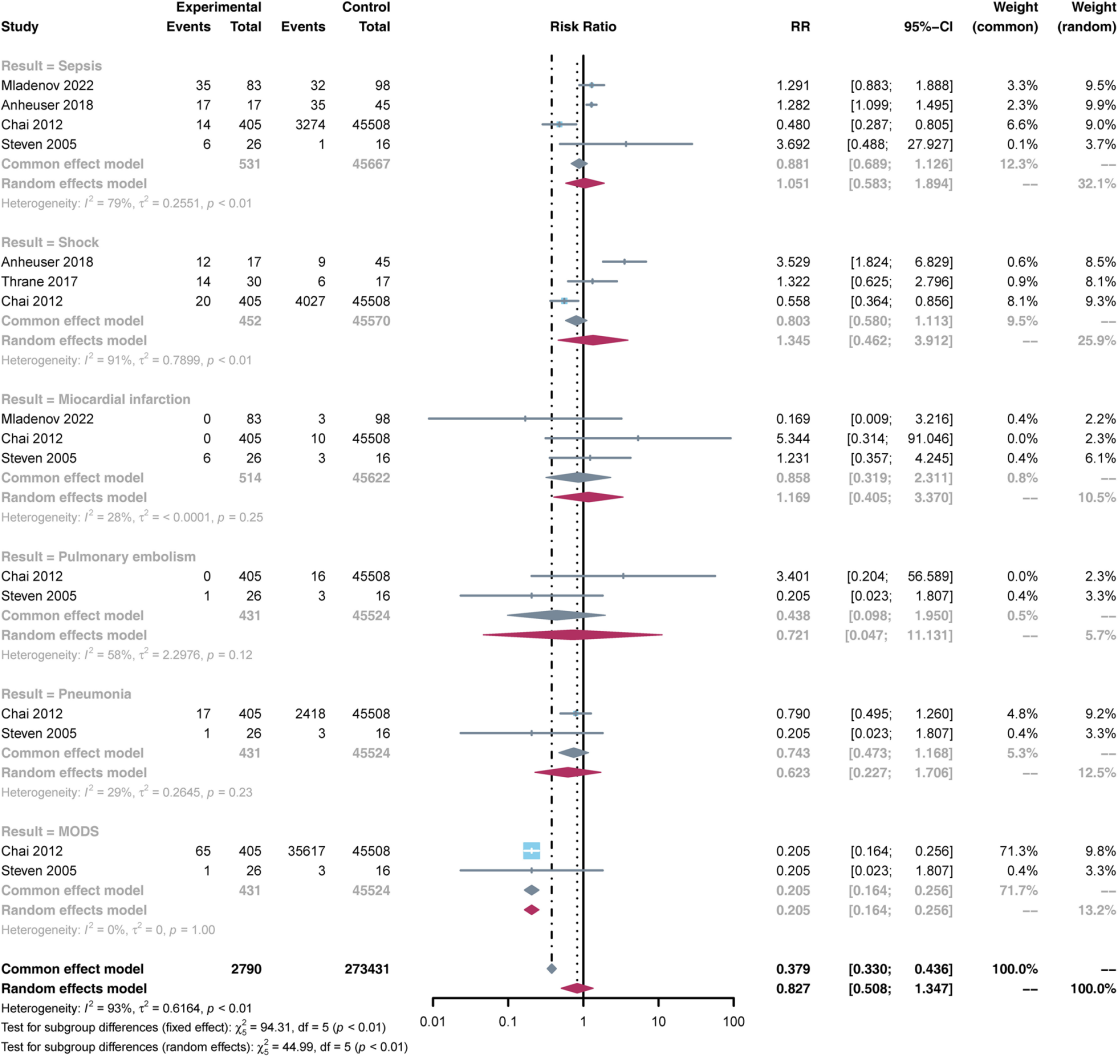
Forest plot of the incidences of complications

Complications were reported in 5 studies [3, 7, 22, 27, 33], including sepsis, shock, myocardial infarction, pulmonary embolism, pneumonia, and MODS. Data on the incidence of MODS was available for analysis from 2 studies [27, 33]. The study found that the incidence of MODS in the HBO group was lower than in the non-HBO group [RR = 0.205, 95% CI (0.164, 0.256), *p* < 0.05]. However, there was no statistical significance in the incidence of other complications, such as sepsis, shock, myocardial infarction, pulmonary embolism, and pneumonia, between the two groups (*p* > 0.05) (Fig. 5).

#### Subgroup analyses

We did a subgroup analysis with pathological entity into two categories: FG subgroup and Non-FG subgroup. The FG subgroup consisted of 10 studies [7, 19–21, 24, 25, 30, 33, 35, 36]. The Non-FG consisted of 13 studies [3, 22, 23, 26–29, 31, 32, 34, 37–39]. The mortality rate was significantly lower in the HBO group compared to the non-HBO group in both non-FG [RR = 0.580, 95% CI (0.436, 0.770), *p* < 0.05] and FG subgroups [RR = 0.389, 95% CI (0.209, 0.723), *p* < 0.05]. The number of debridements in the HBO group was higher than in the non-HBO group [SMD = 0.614, 95% CI (0.453, 0.775), *p* < 0.05] in the non-FG subgroup, while there was no statistical significance of the number of debridements between the HBO group and non-HBO group [SMD = 0.340, 95% CI (− 3.032, 3.712), *p* > 0.05] in the FG subgroup. There was no statistical significance in the incidence rate of sepsis between the HBO group and non-HBO group [RR = 0.800, 95% CI (0.304, 2.108), *p* > 0.05] in the non-FG subgroup, as well as in the FG subgroup [RR = 1.319, 95% CI (0.943, 1.843), *p* > 0.05]. (Table 3). The forest plots of subgroup analyses are showed in Additional file 4.

**Table 3:** Subgroup analysis with pathological entity into two categories

Outcomes	Fournier gangrene (FG subgroup)				Necrotizing soft tissue infection (Non-FG subgroup)			
	No. of studies	No. of particip ants	OR (95%CI)	P for interac tion	No. of studies	No. of particip ants	OR (95%CI)	*P* for interac tion
The mortality rate	10	683	0.389(0.209, 0.723)	0.12	13	48,469	0.580(0.436, 0.770)	0.07
The number of debridements	2	90	0.340(− 3.032, 3.712)	< 0.01	6	758	0.614(0.453, 0.775)	0.71
The incidence of sepsis	2	104	1.319(0.943, 1.843)	0.31	2	46,094	0.800(0.304, 2.108)	< 0.01

*OR*, Odds ratio; *CI*, Confidence interval

#### Publication bias

**Fig. 6:**
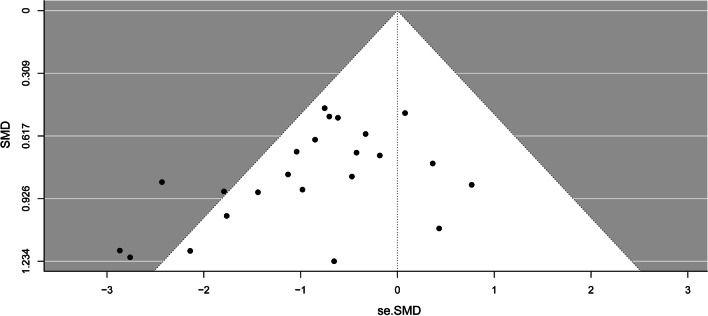
Funnel plot assessing publication bias based on the mortalityrate

The funnel plot did not show significant publication bias for the mortality rate (*p* = 0.086). The funnel plot is shown in Fig. 6.

#### Evidence certainty

The GRADE level of evidence is very low for mortality rate, amputation rate, very low for number of debridement. GRADE evidence certainty for the outcomes is shown in Table 4.

**Table 4:** GRADE evidence certainty

No. of studies	Study design	Risk of bias	Certainty assessment				No. of patients		Effect		Certainty	Importance
			Inconsistency	Indirectness	Imprecision	Other considerations			Relative (95% CI)	Absolute (95% CI)		
*Mortality rate*												
23	Observational studies	Not serious	Not serious	Not serious	Not serious	None	149/1448 (10.3%)	4665/47704 (9.8%)	0.522 – (0.403 to 0.677)	**–**	⨁⨁◯◯ Low	CRITICAL
*Number of debridement*												
8	Observational studies	Not serious	Serious ^a^	Not serious	Not serious	None	523	325	–	0.611 0 (0.012 higher to 1.211 higher)	⨁◯◯◯ Very low	IMPORTANT
*Amputation rate*												
6	Observational studies	Not serious	Not serious	Not serious	Not serious	None	105/836 (12.6%)	6729/45762 (14.7%)	0.836 – (0.619 to 1.129)	–	⨁⨁◯◯ Low	IMPORTANT

^a^represents the high heterogeneity and the cause of the downgrade

*CI*, Confidence interval

## Discussion

NSTI are a type of rapidly progressing infection that can be highly destructive to the skin, subcutaneous tissue, and superficial fascia [40]. These infections involve the breakdown of tissues and fascia and can spread along tissue planes, sometimes resulting in myonecrosis and variable involvement of the skin above [28]. The speed at which the necrotic area progresses is thought to be around 2–3 cm/h [41]. NSTI are also known as NF or FG, and have been referred to as "flesh-eating bug disease." [34] Clinical features of NSTI include grey necrotic tissue, swelling of the fascia, thin, watery, foul-smelling fluid, and blocked vessels [41]. If NSTI is not diagnosed and treated promptly, it can have serious consequences such as limb loss or death [42]. The mortality rate of NSTI has been historically reported to be as high as 76% [43]. Despite advances in diagnostic approaches and treatment regimens, more recent literature has reported mortality rates of NSTI to be between 9 and 25%, or even higher [7]. NSTI often result in local tissue hypoxia. The interaction between tissue hypoxia and infections, along with postoperative incision poor drainage or other factors, can lead to rapid spread of the infection. HBO is a comprehensive treatment that uses a medical hyperbaric oxygen chamber as a carrier and oxygen as the core. Under 0.2 MPa hyperbaric oxygen, physical dissolved oxygen can increase by 17 times, muscle oxygen partial pressure increases by 8 times, and tissue oxygen partial pressure increases by 4 times. Under high pressure, the effective diffusion radius of oxygen extends and the diffusion range expands. HBO has a direct effect on anaerobic bacteria through the formation of oxygen free radicals. During phagocytosis, neutrophil oxygen consumption increases, and HBO can enhance neutrophil activity. HBO can also promote the growth of fibroblasts and the formation of blood vessels, thus promoting wound healing. HBO can alleviate inflammation, reduce inflammatory immune cytokines, stimulate wound repair, maintain wound oxygenation, increase antioxidant enzymes, and treat tissue hypoxemia and radiation necrosis [44–46]. However, there has been ongoing controversy regarding the effectiveness of HBO in terms of mortality and other clinical outcomes in patients with NSTI [25]. Some studies have shown that HBO is significantly beneficial in these patients, while others have found the opposite [28]. To increase the body of evidence, we carried out a systematic review and meta-analysis to compare the outcomes of NSTI patients who were treated with or without HBO.

In this study, 23 eligible retrospective studies were identified, with 65.2% of the patients being male. Previous research has shown that NSTI is more common among elderly males, with a mean age of over 50 years, which is consistent with the findings of this study [47]. The literature reports on the relationship between the incidence of NSTI in patients and gender differences vary, and may be related to the number of cases collected and regional differences. The reason for the different incidence of NSTI between men and women is not yet clear. Zhang et al. [47] reported that this sex difference and age feature may be associated with an increase in the number of conditions that can increase the risk of NSTI. In our study, almost all patients with NSTI had comorbidities, the most common of which were diabetes mellitus, hypertension, alcoholism, smoking, obesity, anorectal diseases, renal disease, malignancy, coronary artery disease, peripheral vascular disease, intravenous drug use, and immunosuppression. Among these predisposing diseases, diabetes mellitus was identified as the most common comorbidity associated with NSTI, which may accelerate bacterial infection progression and result in a poor prognosis, even increasing the risk of mortality [48]. Consistent with the literature, over half of our participants suffered from this comorbidity. High blood sugar is itself a good culture medium for bacteria, and NSTI complicated with diabetes is the result of the combined action of various pathogenic bacteria (aerobic bacteria, anaerobic bacteria, and fungi) [49]. Various pathogenic bacteria can invade the subcutaneous and fascia tissue through the wound. The irritative gases, such as H2, N2, H2S, and CH4, produced by bacteria accumulate in the soft tissue. At the same time, active substances, such as alidase and heparinase, are released to decompose and destroy the tissue, causing corresponding tissue edema and ischemic necrosis [50]. Patients with diabetes are at a higher risk for developing NSTI due to their decreased immune function and increased presence of bacteria on the skin. Diabetic patients also have a decreased ability to phagocytize and a higher potential for local bacterial proliferation, which provides conditions for the proliferation of bacteria. Thus, it is crucial to closely monitor and control blood sugar levels during the treatment of NSTI. It is generally considered that keeping blood sugar levels below 10.0 mmol/L is beneficial for controlling wound infections and granulation growth [51]. Due to the high mortality rate associated with NSTI, we considered mortality to be the primary outcome in this study. Hollabaugh et al. [36] reported a mortality rate of 7% for the HBO group and 42% for the Non-HBO group. Creta et al. [21] reported that mortality due to NSTI occurred in 32 (36.0%) of patients who did not undergo HBO and in 14 (19.4%) of patients who did undergo HBO (*p* = 0.01). Some studies even reported that no patients in the HBO group died [52]. According to the results of this meta-analysis, the mean mortality rate for the HBO group was 10.6% and the mean mortality rate for the Non-HBO group was 25.6%. The mortality rate for the HBO group was significantly lower than that of the Non-HBO group. It is believed that the use of HBO may contribute to this difference by increasing oxygen transport and diffusion to injured, oedematous, and infected hypoxic tissues and by creating a high pressure of oxygen around infected tissue, which can effectively prevent the invasion of microorganisms [16, 23]. Additionally, HBO may narrow the affected region, prevent the extension of necrosis, reduce systemic toxicity, and decrease the mortality rate when used in conjunction with surgical debridement and broad-spectrum antibiotic therapy. The results of this study also showed that the amputation rate was not significantly different between the HBO group and the Non-HBO group. However, it is worth noting that the number of debridements performed in the HBO group was higher than that in the Non-HBO group. Similarly, Tharakaram et al. [53] also observed a higher number of surgical debridements in the HBO group. Usually, debridement surgery has three main purposes, including: to clearly define the extent of infection; to evaluate whether debridement or amputation is necessary; to obtain samples and stain and culture them for bacterial identification. In cases of highly suspected NSTI, performing an effective exploration and sending the sample for pathological and microbiological examination remains the direct evidence for establishing a diagnosis. Therefore, all suspected cases should be promptly explored to establish a clear diagnosis. One study suggested that tissue samples taken for microbiologic analysis were counted as debridement, which may have contributed to the higher number of surgeries in the HBO group due to improved survival [16]. However, further evidence is needed to support this conclusion. It may be inappropriate to use the number of debridements as a measure of the efficacy of HBO. Given the poor prognosis and potential for relapse associated with this disease, the survival rate with HBO based on long-term follow-up should be considered a primary outcome in future meta-analyses. In terms of complications, the results of this study showed that the incidence of MODS was lower in the HBO group than in the Non-HBO group. However, there was no significant difference in the incidence of sepsis, shock, myocardial infarction, pulmonary embolism, or pneumonia between the two groups. These results suggest that the use of HBO is generally safe. HBO can cause oxygen poisoning, sinus barotrauma, middle ear barotrauma or pulmonary barotrauma and other adverse reactions, and induce claustrophobia in severe cases. As a consequence, we should closely monitor all adverse reactions and correct them timely during the treatment of HBO for NSTI. Based on the safety, it is essential to control and regulate the pressure value and time value of HBO, in order to avoid causing serious complications. As FG and NF diseases exhibit distinct epidemiology and pathological characteristics, we conducted a subgroup analysis by categorizing them into two pathological entities. We were limited by the amount of literature available and thus only examined the mortality rate, number of debridements, and incidence rate of sepsis. Among these variables, the number of debridements did not show a significant difference between the HBO and non-HBO groups in the FG subgroup. However, since only two studies were included, further verification is required to confirm the result. Nonetheless, the other subgroup analyses were consistent with previous findings, which suggests the stability of our results.

There are several limitations to be considered in this systematic review and meta-analysis of the effectiveness of HBO in patients with NSTI. Firstly, the retrospective study design may result in inconsistent data quality and availability of certain clinical and laboratory parameters. While this is a limitation, it is worth noting that it would not be ethical to deprive patients of HBO treatment in many cases, and therefore it would not be feasible to conduct a prospective randomized controlled trial [8, 27]. Secondly, the duration and frequency of HBO treatment varied across studies, which could potentially affect the outcomes. It is important to establish unified therapy criteria for HBO in order to ensure consistent treatment. Additionally, the pooled results may be affected by the inclusion of data from different stages of treatment and different diagnostic criteria, courses of treatment, and lengths of follow-up. It is necessary to conduct independent systematic evaluation and analysis according to the different onset sites of NSTI to improve the reliability and stability of outcomes such as mortality rate, amputation rate, and survival rate. Compared with the number of debridement, we think that survival rate should be used as the main outcome indicator for NSTI. Generally, the use of antibiotics in the treatment of NSTI is commonly longer, the scope of debridement is wide, and the healing time is long. Therefore, long-term follow-up of survival patients with NSTI treated with HBO should be carried out to observe the recurrence rate, complications, and quality of life. This should be an important direction for future research. Research on the diagnosis and treatment of NSTI has made some progress in recent years, but the mortality rates and amputation rates of NSTI have not been significantly controlled yet. The main reason is that the progression of NSTI is rapid and early identification is difficult, and the diagnostic criteria are not clear. These are the key points for future research on NSTI.

## Conclusion

The current evidence suggests that the use of HBO in the treatment of NSTI can significantly reduce the mortality rates and the incidence rates of complications. However, due to the retrospective nature of the studies, the evidence is weak, and further research is needed to establish its efficacy. It is also important to note that HBO is not available in all hospitals, and its use should be carefully considered based on the patient's individual circumstances. Additionally, it is still worthwhile to stress the significance of promptly evaluating surgical risks to prevent missing the optimal treatment time. Given the rarity of the disease, it is essential to continue producing high-quality research to provide guidance to clinicians.

## Supplementary Information

**Additional file 1.** PRISMA 2020 Checklist.

**Additional file 2.** PRISMA 2020 for Abstracts Checklist.

**Additional file 3.** The literature search strategy.

**Additional file 4.** Forest plots of subgroup analyses.

## Data Availability

Data for this study are available on request from the corresponding authors.
