# Supplementary material for: The effect of hyperbaric oxygen therapy on the clinical outcomes of necrotizing soft tissue infections: a systematic review and meta-analysis
Source: World J Emerg Surg. 2023 Mar 25;18:23. doi: 10.1186/s13017-023-00490-y (PMC10040118; doi:10.1186/s13017-023-00490-y)
Supplement: Supplementary file 3 — Additional file 3. The literature search strategy. [file 13017_2023_490_MOESM3_ESM.doc]

**Appendix A：**

Search strategy for PubMed from inception to 28^th^, November, 2022.

#1 (Hyperbaric Oxygenations[MeSH Terms]) OR (Oxygenations, Hyperbaric[Title/Abstract]) OR (Hyperbaric Oxygen Therapy[Title/Abstract]) OR (Hyperbaric Oxygen Therapies[Title/Abstract]) OR (Oxygen Therapies, Hyperbaric[Title/Abstract]) OR (Oxygen Therapy, Hyperbaric[Title/Abstract]) OR (Therapies, Hyperbaric Oxygen[Title/Abstract]) OR (Therapy, Hyperbaric Oxygen[Title/Abstract]) OR (Oxygenation, Hyperbaric[Title/Abstract])

#2 (necrotizing soft tissue infection[MeSH Terms]) OR (Fasciitides, Necrotizing[Title/Abstract]) OR (Necrotizing Fasciitides[Title/Abstract]) OR (Necrotizing Fasciitis[Title/Abstract]) OR (Fascitis, Necrotizing[Title/Abstract]) OR (Fascitides, Necrotizing[Title/Abstract]) OR (Necrotizing Fascitides[Title/Abstract]) OR (Necrotizing Fascitis[Title/Abstract]) OR (Gangrene, Fournier[Title/Abstract]) OR (Fournier's Disease[Title/Abstract]) OR (Fourniers Disease[Title/Abstract]) OR (Fournier's Gangrene[Title/Abstract]) OR (Fourniers Gangrene[Title/Abstract]) OR (Gangrene, Fournier's[Title/Abstract]) OR (Fournier Disease[Title/Abstract])

#3 (((review[Title]) OR (meta[Title])) OR (meta-analysis[Title])) OR (review[Publication Type])

#4 #1 AND #2 NOT #3
