# Supplementary material for: The effect of hyperbaric oxygen therapy on the clinical outcomes of necrotizing soft tissue infections: a systematic review and meta-analysis
Source: World J Emerg Surg. 2023 Mar 25;18:23. doi: 10.1186/s13017-023-00490-y (PMC10040118; doi:10.1186/s13017-023-00490-y)
Supplement: Supplementary file 4 — Additional file 4. Forest plots of subgroup analyses. [file 13017_2023_490_MOESM4_ESM.doc]

Forest plots of subgroup analyses
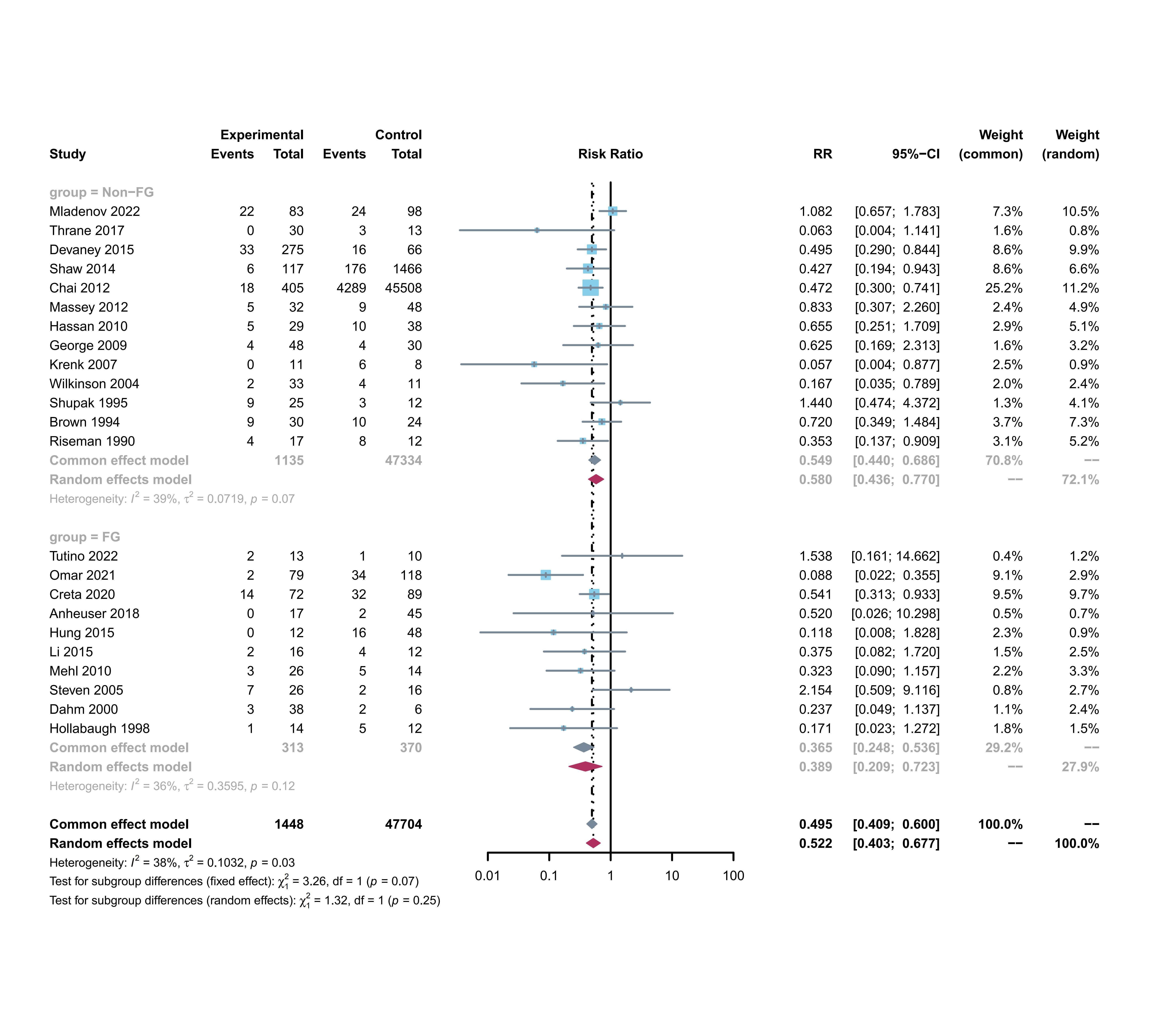
Forest plot of subgroup analysis of the mortality rate


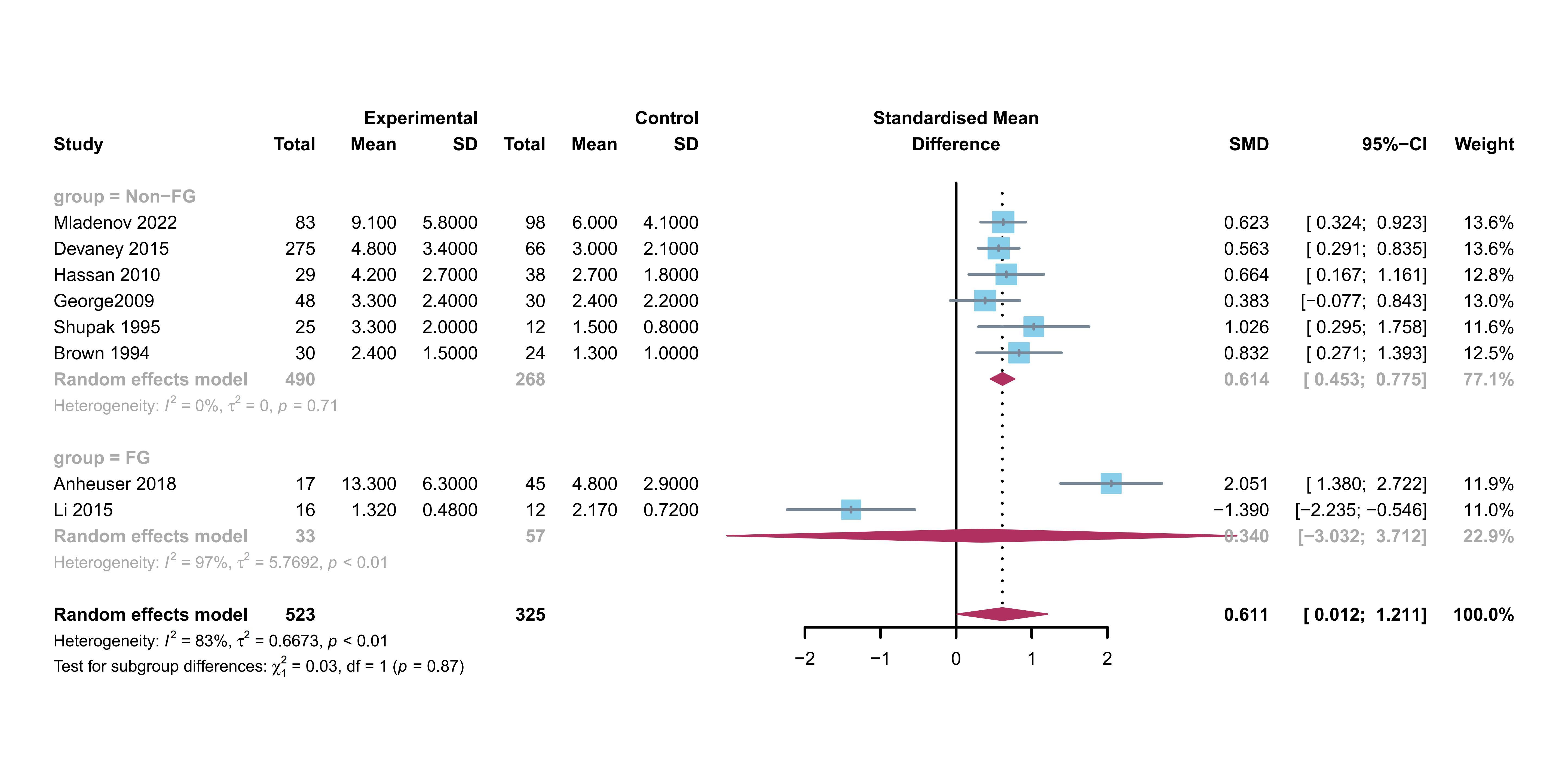


Forest plot of subgroup analysis of the number of debridements


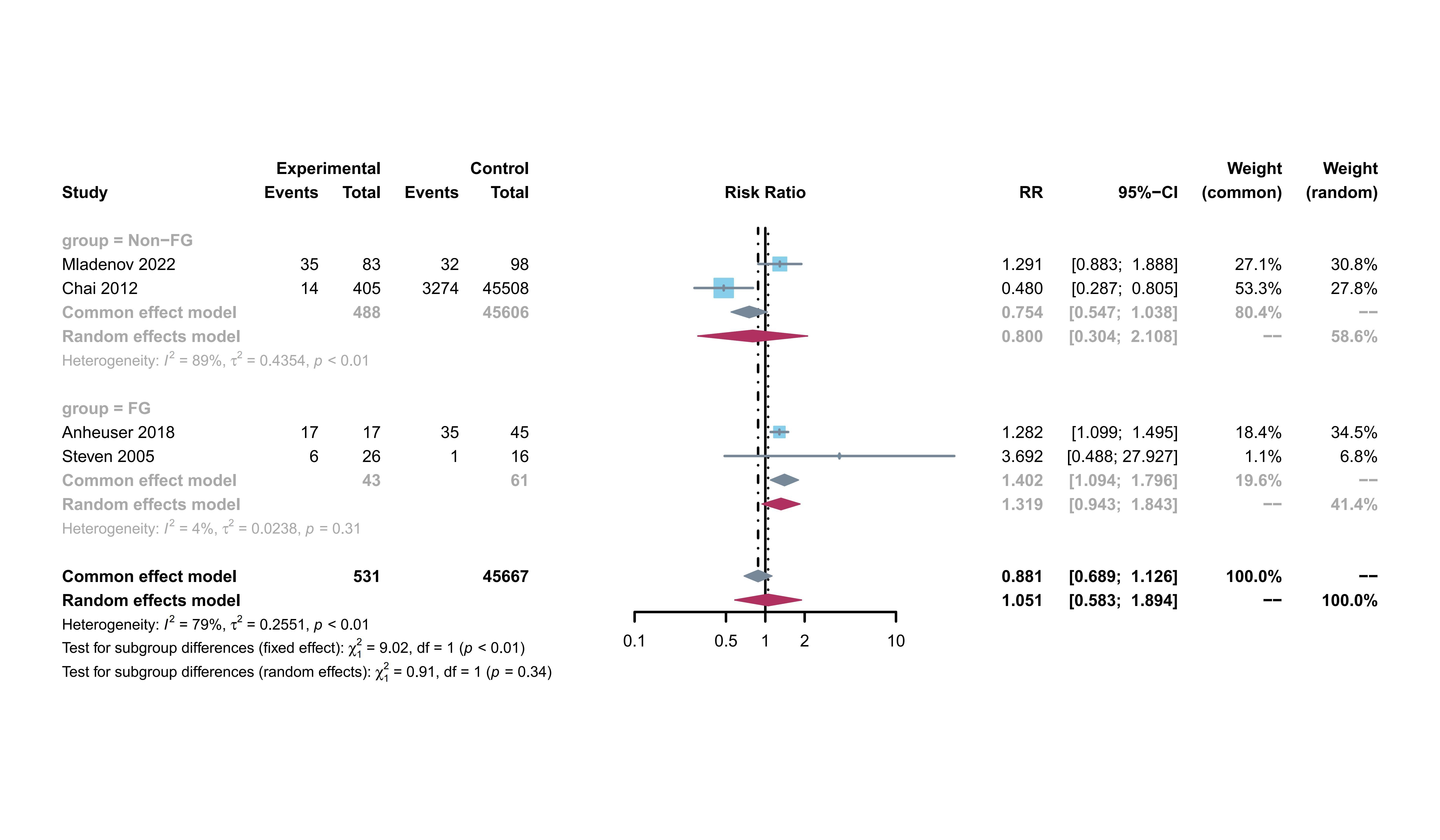


Forest plot of subgroup analysis of the incidence rate of sepsis
